# Supplementary figures and images for: Novel Transcriptional and Translational Biomarkers of Tularemia Vaccine Efficacy in a Mouse Inhalation Model: Proof of Concept
Source: Microorganisms. 2021 Dec 26;10(1):36. doi: 10.3390/microorganisms10010036 (PMC8778127; doi:10.3390/microorganisms10010036)

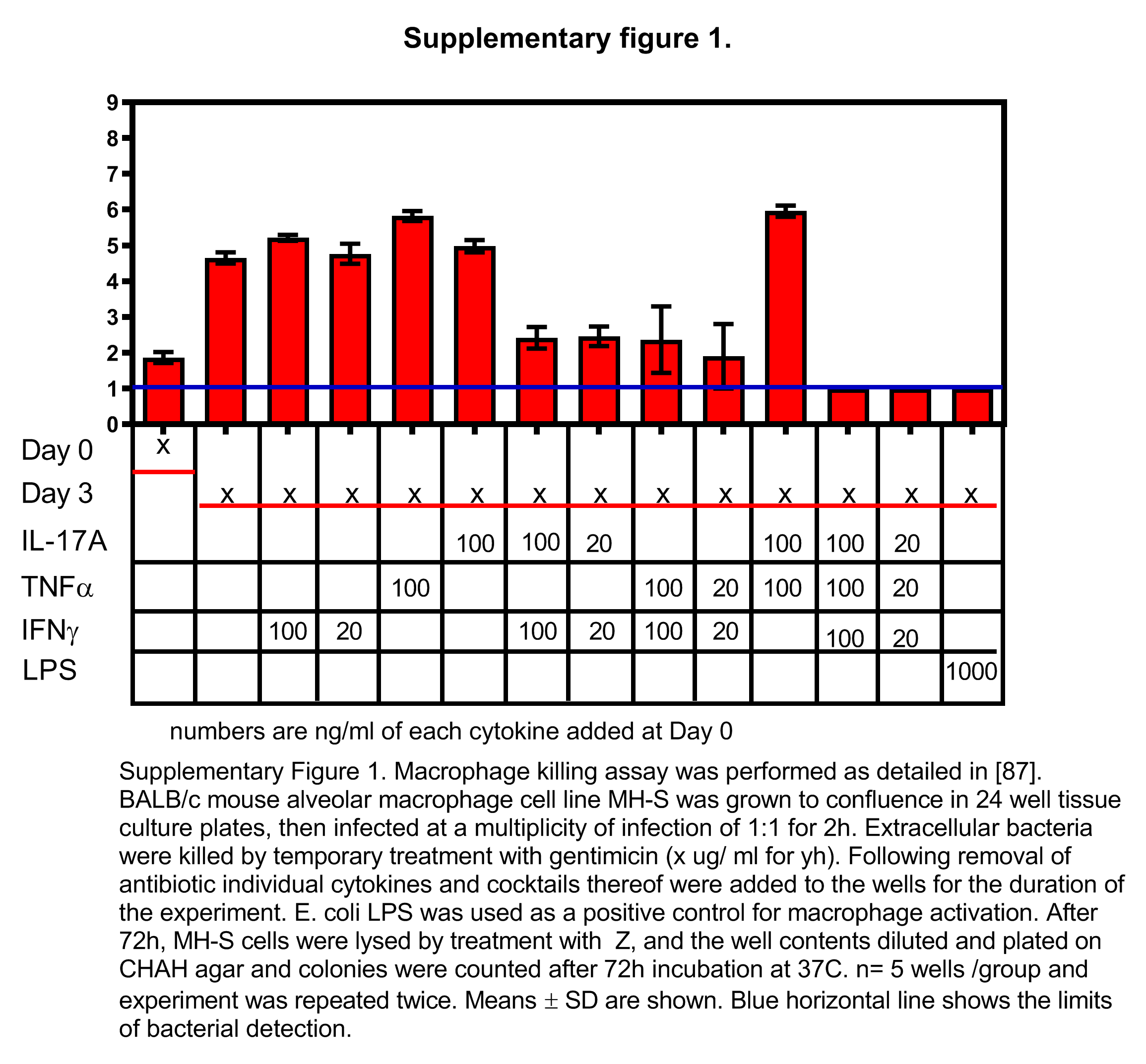

Supplement: Supplementary file 1 [file microorganisms-10-00036-s001.zip › supplementary Figure S1.tif]
